# Evaluation of a retrieval-augmented generation system using a Japanese Institutional Nuclear Medicine Manual and large language model-automated scoring

**DOI:** 10.1007/s12194-025-00941-y

**Published:** 2025-07-19

**Authors:** Yusuke Fukui, Yuhei Kawata, Kazumasa Kobashi, Yukihiro Nagatani, Harumi Iguchi

**Affiliations:** Department of Radiology, Shiga University of Medical Science Hospital, Otsu, Shiga Japan

**Keywords:** Retrieval-augmented generation (RAG), Large language models (LLMs), Nuclear medicine, Medical question answering

## Abstract

Recent advances in large language models (LLMs) enable domain-specific question answering using external knowledge. However, addressing information that is not included in training data remains a challenge, particularly in nuclear medicine, where examination protocols are frequently updated and vary across institutions. In this study, we developed a retrieval-augmented generation (RAG) system using 40 internal manuals from a single Japanese hospital, each corresponding to a different examination in nuclear medicine. These institution-specific documents were segmented and indexed using a hybrid retrieval strategy combining dense vector search (text-embedding-3-small) and sparse keyword search (BM25). GPT-3.5 and GPT-4o were used with the OpenAI application programming interface (API) for response generation. The quality of the generated answers was assessed using a four-point Likert scale by three certified radiological technologists, of which one held an additional certification in nuclear medicine and another held an additional certification in medical physics. Automated evaluation was conducted using RAGAS metrics, including factual correctness and context recall. The GPT-4o model combined with hybrid retrieval achieved the highest performance, as per expert evaluations. Although traditional string-based metrics such as ROUGE and the Levenshtein distance poorly align with human ratings, RAGAS provided consistent rankings across system configurations, despite showing only a modest correlation with manual scores. These findings demonstrate that integrating examination-specific institutional manuals into RAG frameworks can effectively support domain-specific question answering in nuclear medicine. Moreover, LLM-based evaluation methods such as RAGAS may serve as practical tools to complement expert reviews in developing healthcare-oriented artificial intelligence systems.

**Supplementary Information:**

The online version contains supplementary material available at 10.1007/s12194-025-00941-y.

## Introduction

Large language models (LLMs) have demonstrated outstanding performance across a wide range of natural language processing tasks [1–3], and their applications in the medical domain are rapidly expanding [4–6]. However, because LLMs generate responses based on pre-trained data, they have a limited ability to handle domains where information is frequently updated. In practice, fine-tuning LLMs for evolving resources, such as institutional manuals or operational protocols, which are frequently revised at individual healthcare facilities, is technically and operationally difficult.

This limitation is particularly pronounced in nuclear medicine, where examination protocols and procedures continue to diversify owing to the introduction of new radiopharmaceuticals and the spread of theranostics [7, 8]. Institutional manuals often contain detailed descriptions of imaging criteria and procedures that are generally not included in the training data of general-purpose LLMs. As shown in Table 1 (based on our institutional example), these models frequently fail to generate appropriate responses to queries.

**Table 1:** Examples of responses generated by LLM-Only and RAG system at our institution

Item	Content
Question	腎静態シンチグラフィの摂取率の画像作成はどのように行いますか。 [How is the uptake image generated in renal static scintigraphy?]
Answer (LLM-Only [GPT-4o])	腎静態シンチグラフィの摂取率の画像作成は、主に以下の手順で行われます。 1. **放射性医薬品の投与**: 患者に放射性医薬品 (通常はテクネチウム-99m標識化合物) が静脈注射されます。この化合物は腎臓に集まりやすく、腎機能を評価するのに適しています。 2. **撮影準備**: 放射性医薬品が腎臓に集まるまでの時間を待ちます。通常、投与後数分から数十分程度で撮影が可能になります。 3. **ガンマカメラによる撮影**: 患者はガンマカメラの下に横たわり、腎臓から放出されるガンマ線を検出します。ガンマカメラはこれを画像として記録します。 4. **画像処理**: 取得した画像データはコンピュータで処理され、腎臓の形状や放射性医薬品の分布を視覚化します。これにより、腎臓の摂取率や機能を評価することができます。 5. **解析と評価**: 画像を専門医が解析し、腎機能の評価を行います。摂取率の低下や異常な分布が見られる場合は、腎機能の低下や病変の可能性が考えられます。 このプロセスにより、腎臓の機能や形態に関する詳細な情報を得ることができ、診断や治療方針の決定に役立てられます。 [The process of generating uptake images in renal static scintigraphy generally involves the following steps: 1. Administration of radiopharmaceuticals: A radiopharmaceutical, typically a technetium-99 m-labeled compound, is intravenously injected into the patient. This compound tends to accumulate in the kidneys and is suitable for evaluating renal function 2. Preparation for imaging: After administration, time is allowed for the radiopharmaceutical to accumulate in the kidneys. Imaging is typically possible a few to several tens of minutes after injection 3. Imaging with a gamma camera: The patient lies under a gamma camera, which detects the gamma rays emitted from the kidneys. These emissions are recorded as images 4. Image processing: The acquired image data is processed by a computer to visualize the shape of the kidneys and the distribution of the radiopharmaceutical. This allows for assessment of renal uptake and function 5. Analysis and evaluation: A specialist analyzes the images to assess renal function. Decreased uptake or abnormal distribution may indicate impaired renal function or the presence of lesions This process provides detailed information about renal morphology and function and supports diagnostic and therapeutic decision-making.]
Answer (RAG System [GPT-4o/hybrid])	腎静態シンチグラフィの摂取率 (Uptake) 画像作成の手順は以下の通りです: 1. **画像選択** – DMSA、FULL、EMPTY の画像を同時に選択し、[DMSA]アプリケーションを起動します。 2. **患者情報の入力** – 年齢、身長、体重を入力し、[OK]をクリックします。 3. **左腎のROI設定** – 左腎をROI(Region of Interest) で囲み、[Proceed]をクリックします。 ※ 左右を間違えないように注意します。 4. **右腎のROI設定** – 右腎をROIで囲み、[Proceed]をクリックします。 5. **注意事項** – FULLとEMPTYが選択されていなかった場合、この値は算出されません。 – 正常値は20 ~ 25%程度です。 6. **保存** – [Capture Color]を使用して画像を保存します。 以上が摂取率画像作成の手順です。 [Procedure for Creating Uptake Images in Renal Static Scintigraphy (Uptake Calculation): 1. Image Selection Select the DMSA, FULL, and EMPTY images simultaneously, then launch the [DMSA] application 2. Patient Information Entry Enter the patient’s age, height, and weight, and click [OK] 3. Left Kidney ROI Setting Outline the left kidney using the ROI (Region of Interest), then click [Proceed] Note: Be careful not to confuse the left and right sides 4. Right Kidney ROI Setting Outline the right kidney with the ROI, then click [Proceed] 5. Notes If FULL and EMPTY images are not selected, the uptake value will not be calculated The normal range is approximately 20–25% 6. Saving Use [Capture Color] to save the image. This concludes the procedure for generating uptake images]
Reference	DMSA・FULL・EMPTYを同時に選択し、[DMSA]アプリケーション起動します。 年齢・身長・体重を入力し [OK]し、左腎をROIで囲み [Proceed]、右腎をROIで囲み[Proceed]し [Uptake in %Dose]が算出されていることを確認します。 最後にCapture Colorにて保存します。 [Select DMSA, FULL, and EMPTY images simultaneously and launch the [DMSA] application Enter the patient’s age, height, and weight, then click [OK] Outline the left kidney using an ROI and click [Proceed], then outline the right kidney and click [Proceed] again Confirm that [Uptake in %Dose] has been calculated Finally, save the image using Capture Color.]

This table presents examples used in this study, including questions and their corresponding answers generated by the LLM-only and RAG systems implemented at our institution. Reference answers from the evaluation dataset are also shown for comparison. All texts were originally written and processed in Japanese; English translations are provided in brackets [] for reference

Retrieval-augmented generation (RAG) has attracted attention as a promising solution to address this challenge. RAG generates responses by referencing documents retrieved from external sources, making them particularly effective for answering questions that require up-to-date or domain-specific knowledge [9, 10].

In the medical field, RAG has been employed in domains such as nephrology [11], diabetes [12], and preoperative medicine [13]. Some studies have also used institutional documents, not just official clinical guidelines, as sources of retrieved knowledge [13, 14]. However, the usefulness of RAG in the field of radiology has not been thoroughly investigated, and studies involving Japanese-language resources have primarily focused on evaluating standalone LLMs without retrieval mechanisms [15–17].

In this study, we developed a RAG system which used Japanese institutional manuals on nuclear medicine imaging as its knowledge base. The quality of the generated responses was evaluated using a combination of expert ratings—provided by radiological technologists—and automated scoring—by the LLMs. The retrieval component employed a hybrid search approach which combined dense vector retrieval and a sparse keyword-based BM25 algorithm. The generation models used were the GPT-3.5 (OpenAI, San Francisco, CA, USA) and GPT-4o.

This study also aimed to investigate the extent to which automated evaluation by LLMs can approximate or complement expert assessments and provide practical insights into the implementation of LLMs in clinical settings.

## Methods

### Dataset and preprocessing

The knowledge source for the RAG system comprised 40 nuclear medicine imaging manuals in PDF format as of December 2024, which are currently in use at the Department of Radiology, Shiga University of Medical Science Hospital. Each manual contained more than 10,000 tokens, and to optimize both the retrieval and generation performances, the text was split into fixed-length units called chunks.

The chunk size was set to 2000 tokens with an overlap of 1000 tokens. We used RecursiveCharacterTextSplitter from Langchain [18], an open-source framework designed to develop RAG and agent-based applications that integrate LLMs, tools, and memory. Langchain allows for the flexible construction of language-based applications by orchestrating LLMs with external resources such as vector stores and retrievers.

We configured the chunking process to prioritize splitting at natural linguistic boundaries such as spaces, line breaks, and punctuation marks, including Japanese commas (“、”) and periods (“。”), thereby avoiding cutting off sentences midway, while keeping each chunk within the 2000-token limit. The chunking process was designed to ensure that the description of a single application remained within a single chunk. Because the examination name appeared only at the beginning of each manual, a header sentence such as “The following is a part of the manual for the examination named ‘XXX’” was added to the beginning of each chunk. One of the nuclear medicine imaging manuals used as an external knowledge source in this study, the brain perfusion scan manual, is provided as an example in the Supplementary Material.

For the evaluation, we used a dataset consisting of 100 manually created question–answer pairs designed by certified radiological technologists and medical physicists with practical experience. Each question was reviewed by two certified nuclear medicine technologists to ensure clinical relevance. The full list of questions–reference answer pairs created for the evaluation of this RAG system is presented in Supplementary Table S1.

### System configuration

**Fig. 1:**
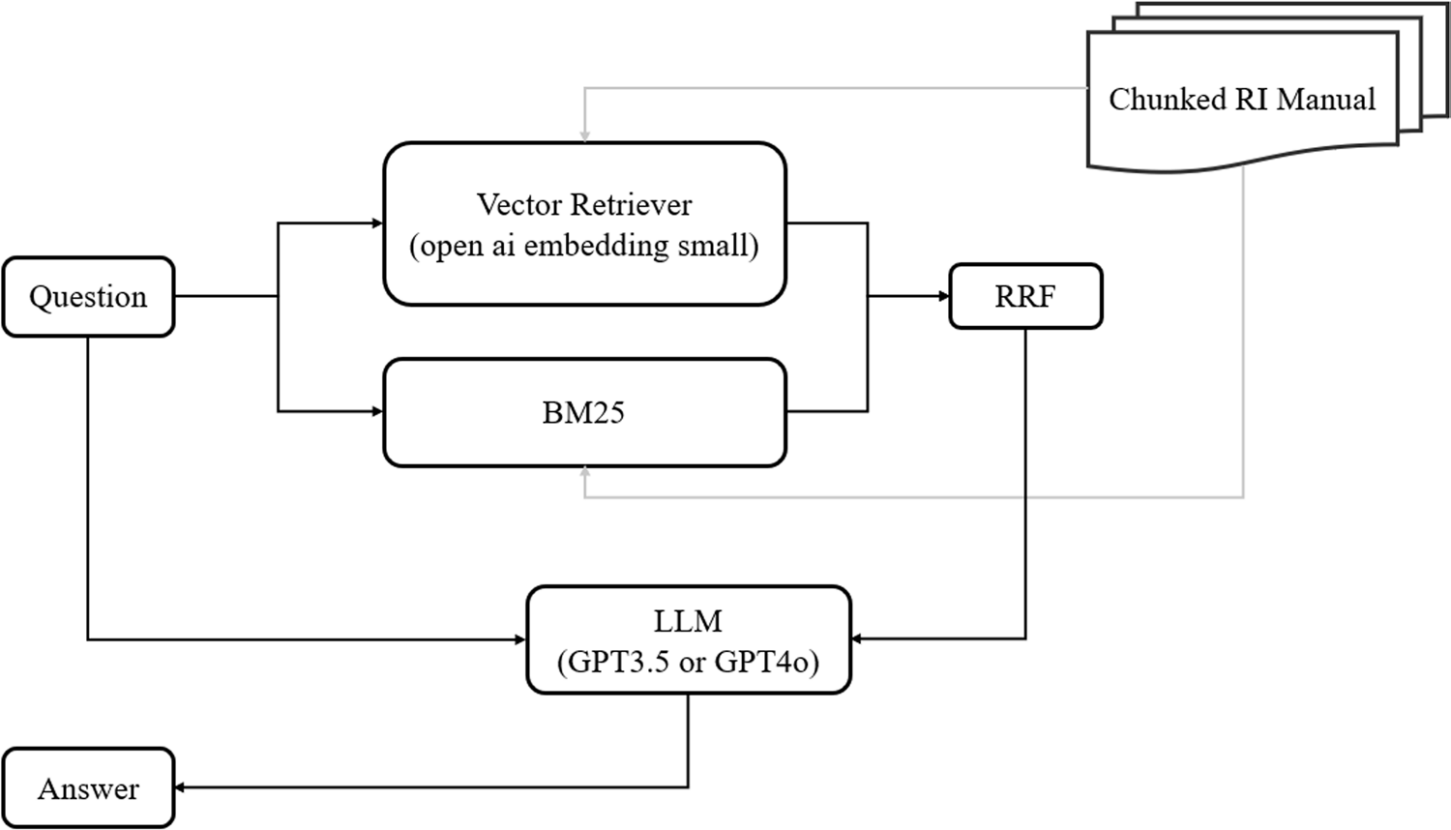
Overview of the RAG system. Overview of the RAG system developed in this study showing how a user question is sent in parallel to a dense vector retriever (OpenAI text-embedding-3-small) and a sparse BM25 retriever, how the passages retrieved from the chunked RI manual are merged by reciprocal rank fusion (RRF), and how the fused contexts together with the original question are provided to an LLM (GPT-3.5 or GPT-4o) to generate the final Answer; boxes represent system components and arrows indicate the direction of data flow

We implemented a Japanese-language RAG system using institutional manuals as a knowledge base and compared multiple system configurations. Two retrieval mechanisms were tested: (1) dense vector retrieval alone, and (2) hybrid retrieval combining a keyword search (BM25) and dense vector search. Each RAG system was defined as a combination of the document chunks retrieved by each method and the LLM-based answer generation module described below. An overview of the RAG system with hybrid retrieval is shown in Fig. 1.

For dense retrieval, we used OpenAI’s text-embedding-3-small model to compute the vector embeddings of document chunks. The top five chunks were selected based on their cosine similarity to the query.

For hybrid retrieval, in addition to vector search, we applied keyword-based retrieval using the BM25 algorithm. As part of this keyword retrieval process, a Japanese morphological analysis was conducted using SudachiPy [19] to segment the text, and document-level relevance scores were calculated accordingly. The top five results from both retrieval methods were merged, and the top five documents were selected using reciprocal rank fusion (RRF).

For response generation, we used GPT-3.5 and GPT-4o as foundation models via the OpenAI API. The query and retrieved document chunks were combined into a prompt, and generation was performed with the temperature fixed at 0 for consistency. Four RAG configurations (two retrieval methods × two generation models) were evaluated. The structure of the prompt is as follows, where {context} is replaced by the retrieved results and {question} is replaced by the input question.

Prompt Template

*Please answer the following question based only on the provided context.*

*Context:*

*"""*

*{context}*

*"""*

*Question: {question}*

Note: In the actual implementation, all prompts were issued in Japanese.

All prompts were submitted to the OpenAI API between 11 December 2024 and 9 January 2025, using fixed snapshot versions of each model (e.g. gpt-4o-2024-11-20) throughout the study. The details of the Python libraries and LLM configurations used in this study are listed in Supplementary Tables S2 and S3, respectively. All processes were executed using the OpenAI API in Python 3.11.12.

### Evaluation metrics

#### Human evaluation

The generated responses were evaluated by two radiological technologists (one with expertise in nuclear medicine) and a medical physicist. The radiological technologists had 25 years (certified nuclear medicine technologist) and 5 years of experience in nuclear medicine, respectively, while the medical physicist had 15 years of experience. This diversity was intended to ensure a multiperspective evaluation. The radiological technologist with 5 years of experience was also involved in the creation of the dataset, which may have introduced potential bias in the evaluation. To mitigate this, all evaluators reviewed all question–reference answer pairs in the dataset prior to assessing the RAG system outputs. During the evaluation, they referred not only to the questions but also to the reference answers, alongside the RAG-generated responses. The evaluators also had access to the institutional manual throughout the evaluation process. A four-point Likert scale was used to assess the quality of the responses.

- (4) Ideal answer
- (3) Appropriate but verbose
- (2) Partially incomplete
- (1) Inappropriate

The scores were normalized to a range of 0–1 and averaged across the raters. The retrieved documents were evaluated using a similar four-point scale:

- (4) Mostly optimal
- (3) Generally relevant
- (2) Partially relevant
- (1) Irrelevant

#### Automated evaluation

We used RAGAS (v0.2.8) for the LLM-based automated evaluation [20]. The scoring models were GPT-3.5 or GPT-4o-mini, with the temperature set to 0. Due to the possibility of interruptions caused by the rate limits of the OpenAI API during evaluation, we used GPT-4o-mini for stable execution and conducted a comparative evaluation with GPT-3.5.

We employed the following RAGAS metrics:

- Factual correctness: The degree to which the generated answer aligns with the reference answer in terms of factual content. This metric evaluates whether the key facts and claims in the generated response are accurate and supported by the source information. It focuses on factual accuracy rather than surface similarity or fluency.
- Context recall: The degree to which retrieved documents (chunks) contain the information necessary to construct a reference answer. In this study, “context” refers to the top-ranked documents retrieved, and context recall evaluates their coverage from the perspective of completeness.

Additionally, we included traditional metrics as supplementary indicators:

- Semantic similarity: Cosine similarity between sentence embeddings computed by text-embedding-3-small. While similar to BERTScore in measuring semantic consistency, this metric differs in that BERTScore performs token-level scoring, whereas semantic similarity provides a single scalar value based on the cosine similarity of sentence-level embeddings.
- ROUGE score: Calculated based on n-gram overlap after Japanese morphological analysis (SudachiPy). This metric evaluates how well the generated responses cover the reference answers.
- Levenshtein distance: Edit distance between character strings. This metric calculates the number of character-level edits required to transform the generated text into the reference answer, thereby evaluating the structural similarity between the two texts.

### Statistical analysis

To assess the agreement between the human and automated scores, we computed Pearson correlation coefficients for each RAG configuration. Differences in the evaluation scores across the generation models and retrieval methods were analyzed using the Wilcoxon rank-sum test. For each evaluation metric, pair-wise comparisons between system outputs were conducted using the two-sided Wilcoxon signed-rank test with zero_method = "zsplit", which splits zero differences evenly between positive and negative ranks. The test statistic W was defined as the smaller of the summed positive and negative ranks. Z-scores were obtained from the normal approximation,

$$Z=\left(W-\mu \right)/\alpha ,\hspace{0.25em}\hspace{0.25em}\text{with}\hspace{0.25em}\hspace{0.25em}\mu =n(n+1)/4,\hspace{0.25em}\hspace{0.25em}\text{and}\hspace{0.25em}\hspace{0.25em}\alpha =\sqrt{\left[n(n+1)(2n+1)/24\right]},$$

where *n* is the total number of observation pairs included in the comparison. The effect size was calculated as $r=\left|Z\right|/\surd n.$

To further examine differences attributable to the nature of the questions, we provided GPT-4o with the following classification criteria as a prompt and asked it to label each question accordingly: S (Standard application)—explanations of standard procedures or protocols explicitly described in the manual; K (Keyword-based factual recall)—single factual items such as the name of a radiopharmaceutical, imaging timing, or equipment; and A (Analytical reasoning)—requests that require contextual processing beyond a single answer, such as positioning instructions or image lists. Differences across these question categories were tested for significance using the Kruskal–Wallis test. The category assigned to every question–answer pair is listed together with the full list in Supplementary Table S1.

To assess potential rater effects, evaluator scores—treated as paired data—were analyzed with the Friedman test; when significant, post-hoc Wilcoxon signed-rank tests with Holm correction were performed.

In all statistical analyses, statistical significance was defined as *p* < 0.01.

## Results

### Human evaluation of generated answers

Table 2 lists the average human evaluation scores for the answers generated for each RAG configuration. The combination of GPT-4o and hybrid retrieval achieved the highest score of 84.0 (out of 100). This was followed by GPT-4o with dense retrieval (81.33), GPT-3.5 with hybrid retrieval (69.67), and GPT-3.5 with dense retrieval (69.17).

**Table 2:** Evaluation scores by the retrieval method and generation model

	Hybrid/3.5	Hybrid /4o	Vector/3.5	Vector/4o
Human as a Judge				
Total score answer	69.67	84.00	69.17	81.33
Total score retrieve	56.33	56.33	49.17	49.17
LLM as a Judge (3.5)				
Factual correctness	52.51	67.66	49.27	65.66
Context recall	86.87	86.87	85.70	85.70
LLM as a Judge (4o-mini)				
Factual correctness	41.80	51.26	40.96	46.25
Context recall	84.92	84.92	75.92	75.92
Other evaluation				
Semantic similarity	62.74	57.17	59.51	55.89
ROUGE score	46.52	40.14	44.86	38.59
Levenshtein distance	31.47	20.33	29.99	18.86

Note. Because both generation models used identical retrieval procedures and hyperparameters, the retrieved contexts were identical. Consequently, the Total score retrieve and Context recall values are the same for both models. For clarity, these identical values are reported in both model columns

The difference in scores between the generation models was statistically significant, and GPT-4o consistently received higher ratings regardless of the retrieval method used. Details of the statistical comparisons between the configurations are provided in Table 3.

**Table 3:** Comparison between RAG systems and statistical analysis results

		System1 name: System2 name	Total score (System1)	Total score (System2)	W statistic	*p* value	Effect size *r*
Human as a Judge	Total score (answer)	Hybrid/4o: Hybrid/3.5	84.00	69.67	1318.0	*p* < 0.001	0.419
		Vector/4o: Vector/3.5	81.33	69.17	1717.5	*p* < 0.001	0.278
		Hybrid/3.5: Vector/3.5	69.67	69.17	2453.0	*p* = 0.801	0.025
		Hybrid/4o: Vector/4o	84.00	81.33	2319.0	*p* = 0.475	0.070
	Total score (retrieve)	Hybrid/ Vector	56.33	49.17	1306.5	*p* < 0.001	0.419
LLM as a Judge (GPT-3.5)	Factual correctness	Hybrid/4o: Hybrid/3.5	67.66	52.51	1321.5	*p* < 0.001	0.295
		Vector/4o: Vector/3.5	65.66	49.27	1063.5	*p* < 0.001	0.495
		Hybrid/3.5: Vector/3.5	52.51	49.27	2134.0	*p* = 0.175	0.134
		Hybrid/4o: Vector/4o	67.66	65.66	1554.5	*p* = 0.066	0.194
	Context recall	Hybrid/ Vector	86.87	85.70	2364.5	*p* = 0.554	0.019
LLM as a Judge (GPT-4o-mini)	Factual correctness	Hybrid/4o: Hybrid/3.5	51.26	41.80	1908.5	*p* = 0.033	0.212
		Vector/4o: Vector/3.5	46.25	40.96	2169.5	*p* = 0.220	0.122
		Hybrid/3.5: Vector/3.5	41.80	40.96	2390.5	*p* = 0.635	0.046
		Hybrid/4o: Vector/4o	51.26	46.25	2133.0	*p* = 0.173	0.135
	Context recall	Hybrid/ Vector	84.92	75.92	2235.0	*p* = 0.271	0.100
Other evaluation	Semantic similarity	Hybrid/4o: Hybrid/3.5	57.17	62.74	1335.0	*p* < 0.001	0.409
		Vector/4o: Vector/3.5	55.89	59.51	1508.0	*p* < 0.001	0.349
		Hybrid/3.5: Vector/3.5	62.74	59.51	1680.0	*p* < 0.01	0.290
		Hybrid/4o: Vector/4o	57.17	55.89	1806.0	*p* = 0.013	0.247
	ROUGE score	Hybrid/4o: Hybrid/3.5	40.14	46.52	1548.0	*p* < 0.001	0.336
		Vector/4o: Vector/3.5	38.59	44.86	1569.0	*p* < 0.001	0.328
		Hybrid/3.5: Vector/3.5	46.52	44.86	2229.5	*p* = 0.291	0.101
		Hybrid/4o: Vector/4o	40.14	38.59	2444.0	*p* = 0.774	0.028
	Levenshtein distance	Hybrid/4o: Hybrid/3.5	20.33	31.47	673.5	*p* < 0.001	0.576
		Vector/4o: Vector/3.5	18.86	29.99	667.0	*p* < 0.001	0.638
		Hybrid/3.5: Vector/3.5	31.47	29.99	2224.0	*p* = 0.299	0.103
		Hybrid/4o: Vector/4o	20.33	18.86	2020.5	*p* = 0.083	0.173

This table summarizes the results of pairwise comparisons between different RAG systems, combining Hybrid or Vector-based retrieval with GPT-4o or GPT-3.5

Evaluations were conducted by human annotators (Human as a Judge) and large language models (LLM as a Judge, GPT-3.5 and GPT-4o-mini), using metrics such as total answer score, retrieval score, factual correctness, context recall, Levenshtein distance, semantic similarity, and ROUGE score

To assess statistical significance between paired conditions, we applied a two-sided Wilcoxon signed-rank test with zero_method = "zsplit"; the resulting *W* statistic, *p*-value, and effect size are reported for each comparison

### Results of automated evaluation

For the RAGAS metrics, GPT-4o outputs generally yielded the highest factual correctness scores. However, traditional automated metrics, such as ROUGE and Levenshtein distance, showed higher scores for GPT-3.5 displaying trends opposite to those observed in human evaluations.

### Evaluation of retriever performance

In terms of retrieval performance alone, hybrid retrieval received significantly higher ratings than dense vector retrieval. This may be attributed to the compatibility of the BM25-based keyword matching with the characteristics of medical terminology.

However, answer accuracy showed no statistically significant differences between the retrieval methods. Therefore, the quality of the retriever does not necessarily translate directly into the accuracy of the generated responses.

The average human rating for the retrieved context was 50 out of 100, suggesting that nearly half the retrieved chunks contained some degree of irrelevant information or noise. However, the LLM was able to extract appropriate information from noisy results and generate correct answers, rated as either (4) ideal or (3) appropriate but verbose, for approximately 80% of the questions.

### Correlation between human and automated evaluations

Tables 4 and 5 show the Pearson correlation coefficients between the human ratings and automated metrics. In Table 4, the answers generated by GPT-3.5 showed consistently higher correlations with human ratings than those generated by GPT-4o. This pattern was observed in both the hybrid and dense retrieval settings. As shown in Table 5, the correlations for retrieval quality (context recall) were generally low. However, scores evaluated using GPT-4o-mini showed slightly higher correlations than those evaluated using GPT-3.5, especially in the dense retrieval setting (*r* = 0.458). Overall, the correlations were weak to moderate, indicating limited agreement between human and automated evaluations.

**Table 4:** Correlation between human and LLM evaluations of answer quality

	Factual correctness (3.5)	Factual correctness (4o-mini)	Semantic similarity	ROUGE score	Levenshtein distance
Total score answer (Hybrid/3.5)	0.535	0.501	0.382	0.500	0.489
Total score answer (Hybrid/4o)	0.262	0.188	0.263	0.262	0.332
Total score answer (Vector /3.5)	0.612	0.547	0.494	0.525	0.481
Total score answer (Vector /4o)	0.325	0.310	0.391	0.290	0.374

**Table 5:** Correlations between human ratings and LLM-based evaluations of retrieval performance

	Context recall (3.5)	Context recall (4o-mini)
Total score retrieve (Hybrid)	0.097	0.130
Total score retrieve (Vector)	0.200	0.458

### Statistical differences in evaluation by question categories and evaluators

**Fig. 2:**
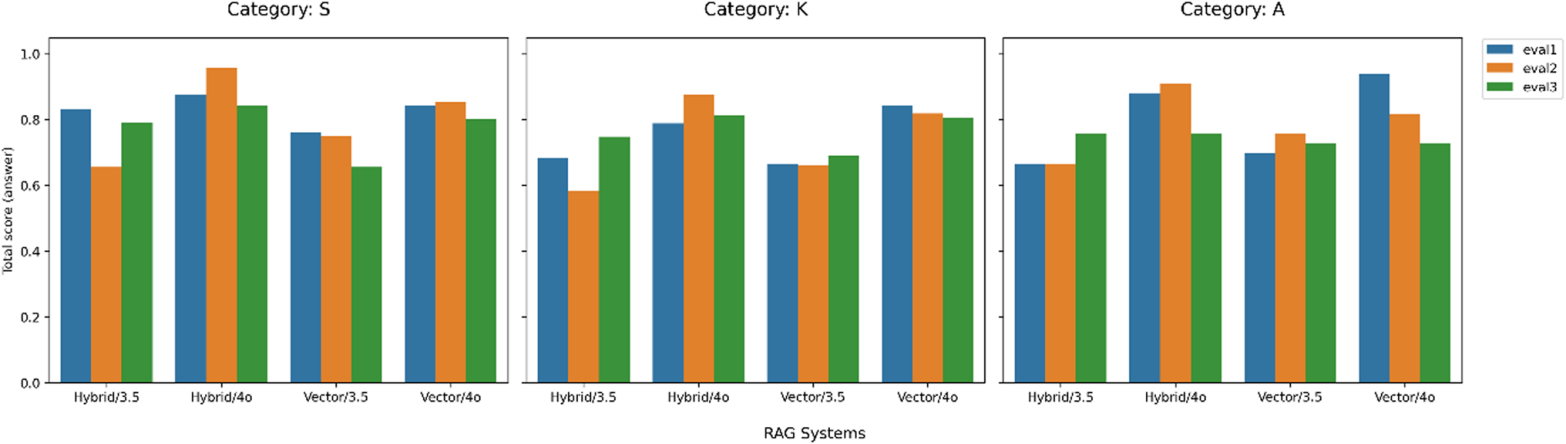
Average scores by RAG systems and evaluators across categories. Average scores assigned by three evaluators to four RAG systems, separated by question category—S (Standard application), K (Keyword-based factual recall), and A (Analytical reasoning); panels (**a**)–(**c**) correspond to categories S, K, and A, respectively, with bars grouped by system and color-coded by evaluator

Figure 2 presents the mean scores for each system–evaluator combination, stratified by question category. Evaluator 1 was a certified nuclear medicine technologist with 25 years of experience, Evaluator 2 was a radiological technologist with five years of nuclear-medicine experience who also helped create the questions, and Evaluator 3 was a medical physicist with 15 years of nuclear-medicine experience. Although some variability is evident in the evaluations, no tendency for any specific evaluator to systematically assign higher or lower scores was observed. Table 6 shows the evaluation results by question category, and Table 7 shows the evaluation results by evaluator. No significant differences were found between question categories across all RAG systems, suggesting that the type of question did not affect answer accuracy within the context of questions related to clinical nuclear medicine procedures. Differences in evaluator scores were not significant for vector-based retrieval but were significant for hybrid retrieval. For the hybrid retrieval condition, we conducted Wilcoxon signed-rank tests with Holm correction, and the results are shown in Supplementary Tables S4 and S5. These results indicated no significant difference between the medical physicist and the certified nuclear medicine technologist, but significant differences were found between these two and the other radiological technologist.

**Table 6:** Statistical comparison of evaluation scores among question categories

	*H* (Statistic)	*p*-value	*ε*^2^ (Effect size)
Total score answer (Hybrid/3.5)	*H* = 0.511	*p* = 0.759	0
Total score answer (Hybrid/4o)	*H* = 0.180	*p* = 0.913	0
Total score answer (Vector /3.5)	*H* = 0.354	*p* = 0.837	0
Total score answer (Vector /4o)	*H* = 1.057	*p* = 0.589	0

This table presents the results of the Kruskal–Wallis *H* test, which examined whether the median evaluation scores differed across question categories (K, A, S). The accompanying effect size (*ε*^2^) was computed with the Tomczak and Tomczak [27] approximation and clipped to 0 when negative. No statistically significant differences were observed among the categories

**Table 7:** Statistical comparison of evaluation scores among evaluators

	Friedman statistic	*p*-value	Kendall’s *W*
Total score answer (Hybrid/3.5)	*χ*^2^ = 23.066	*p* < 0.001	0.217
Total score answer (Hybrid/4o)	*χ*^2^ = 16.466	*p* < 0.001	0.149
Total score answer (Vector /3.5)	*χ*^2^ = 0.747	*p* = 0.688	0.004
Total score answer (Vector /4o)	*χ*^2^ = 6.827	*p* = 0.033	0.050

The Friedman test was applied because three raters repeatedly scored the same set of questions for each RAG system configuration. Alongside the test statistic (*χ*^2^) and *p*-value, we report Kendall’s *W* as an effect-size measure of inter-rater disagreement

### Failure cases and error analysis

Even with the best-performing configuration (GPT-4o + hybrid retrieval), the human-rated answer score was low in some instances (normalized score ≤ 0.333). These cases were attributed to missing information in the manuals or the failure of the retriever to retrieve relevant documents. In particular, abstract questions spanning multiple examinations led to poor retrieval performance, which negatively affected answer accuracy. Representative examples of these failures are listed in Table 8.

**Table 8:** Examples of low-quality answers generated by the hybrid retriever with GPT-4o and possible causes

Question	Total score answer	Reasons for poor answer
99mTc-TFを用いた心筋血流の負荷検査で送信する画像をおしえてください。 [What images should be sent in a myocardial perfusion stress testing using 99mTc-TF?]	0.167	Although the RI exam manuals contain information relevant to the question, the retrieval module failed to retrieve it, which may have led to an inadequate response
ヨード甲状腺シンチグラフィの検査前に摂取してはいけないものはなんですか。 [What should I avoid consuming before an iodine thyroid scan?]	0.167	The RI exam manuals did not contain any information relevant to the question
当院でInを使用する検査は何がありますか。 [What studies at our hospital use indium (In)?]	0.333	Although several documents were required to fully address the question, only a subset of them was retrieved, which may have led to an incomplete response
心筋血流シンチグラフィの前処置について説明してください。 [Could you explain the preparation procedures for myocardial perfusion scintigraphy?]	0.000	Although several documents were required to fully address the question, only a subset of them was retrieved, which may have led to an incomplete response
甲状腺シンチで、亜急性甲状腺炎の臨床像はどうようになりますか。 [What is the clinical appearance of subacute thyroiditis on a thyroid scintigraphy?]	0.167	The RI exam manuals did not contain any information relevant to the question
肝受容体シンチグラフィの定量値であるHH15・LHL15の正常値を教えてください。 [What are the normal values for HH15 and LHL15, which are quantitative values in liver receptor scintigraphy?]	0.000	Although relevant information was present in the RI exam manuals, it was provided solely in the form of a figure, limiting its usability for the system
脳血流検査では何MBqの薬剤を使用しますか。 [How many MBq of radiopharmaceutical is used in a cerebral blood flow study?]	0.333	Although several documents were required to fully address the question, only a subset of them was retrieved, which may have led to an incomplete response
薬剤のpre撮像が必要な検査は何ですか。 [Which studies require pre-administration imaging?]	0.333	Although several documents were required to fully address the question, only a subset of them was retrieved, which may have led to an incomplete response

This table presents representative examples of low-quality answers generated by GPT-4o when used with a hybrid retriever. All content was originally written in Japanese, with English translations [] provided for reference. Responses were evaluated by two radiological technologists (one specializing in nuclear medicine) and a medical physicist, using a four-point Likert scale: (4) Ideal answer, (3) Appropriate but verbose, (2) Partially incomplete, (1) Inappropriate. Scores were normalized to a 0–1 range and averaged across raters

The table includes three columns: Question, Total score Answer, and Reasons for poor answer. “Question” lists the input prompts; “Total score answer” shows the average normalized score based on human ratings; and “Reasons for poor answer” describes the likely causes of reduced answer quality, such as retrieval failure, insufficient content in the reference manual, or unstructured input formats (e.g., figures or tables), which may have led to hallucinations or incomplete responses

### Observation of hallucinations

As shown in Table 9, at least one human evaluator rated the retrieved contexts as irrelevant in several instances. Two such occurrences were observed with hybrid retrieval and five with dense retrieval. In these scenarios, GPT-4o often responded cautiously by explicitly stating that it could not answer the question because of insufficient information. In contrast, GPT-3.5 tended to generate incorrect or fabricated content, the so-called hallucinations.

**Table 9:** Examples of answers with low retrieval-relevance scores

	Total score answer	Factual correctness (3.5)	Factual correctness (4o-mini)	Semantic similarity	Levenshtein distance	ROUGE score
Hybrid Retriever—Example 1						
Score (3.5)	0.000	0.500	0.000	0.618	0.195	0.000
Score (4o)	0.111	0.670	0.570	0.516	0.083	0.000
Question	甲状腺シンチで、亜急性甲状腺炎の臨床像はどのようになりますか。					
	[What is the clinical presentation of subacute thyroiditis on a thyroid scintigraphy?]					
Reference	亜急性甲状腺炎では集積が見られないことがほとんどです。					
	[In subacute thyroiditis, radiotracer uptake is rarely observed.]					
Answer (3.5)	甲状腺シンチでは、亜急性甲状腺炎の臨床像は通常、甲状腺の局所的な炎症や腫脹が観察されます。炎症が進行すると、甲状腺の機能が一時的に亢進することがあります。					
	[In thyroid scintigraphy, the clinical presentation of subacute thyroiditis typically includes localized inflammation and swelling of the thyroid gland. As the inflammation progresses, transient hyperthyroidism may occur.]					
Answer (4o)	文脈に基づいて回答します。 提供された文脈には、甲状腺シンチ (201Tl) に関する検査手順や撮像条件についての情報が記載されていますが、亜急性甲状腺炎の臨床像やその診断に関する具体的な記載はありません。そのため、文脈内の情報だけでは亜急性甲状腺炎の臨床像について回答することはできません。					
	[Based on the provided context, which includes details about the examination procedure and imaging conditions for thyroid scintigraphy (201Tl), there is no specific information regarding the clinical presentation or diagnosis of subacute thyroiditis. Therefore, it is not possible to answer the question based solely on the given context.]					
Hybrid Retriever—Example 2						
Score (3.5)	0.222	0.670	0.670	0.609	0.467	0.727
Score (4o)	0.778	1.000	0.548	0.548	0.091	0.556
Question	血糖値のmg/dLからmmol/Lへの換算係数を教えてください。					
	[What is the conversion factor for blood glucose levels from mg/dL to mmol/L?]					
Reference	18 mg/dL = 1 mmol/Lです。					
	[18 mg/dL = 1 mmol/L.]					
Answer (3.5)	血糖値のmg/dLからmmol/Lへの換算係数は、18です。					
	[To convert blood glucose levels from mg/dL to mmol/L, divide by 18.]					
Answer (4o)	文脈に血糖値の換算係数に関する情報は含まれていません。そのため、文脈外の一般的な知識としてお答えします。 血糖値をmg/dLからmmol/Lに換算するには、値を**18で割ります**。 例えば、100 mg/dLは約5.56 mmol/Lになります。 もし文脈に関連する情報が必要であれば、具体的な箇所を教えてください。					
	[The provided context does not contain information about blood glucose conversion factors, so the following is based on general medical knowledge. To convert blood glucose from mg/dL to mmol/L, divide the value by 18. For example, 100 mg/dL is approximately 5.56 mmol/L. If you need information specifically related to the context, please indicate the relevant section.]					
Vector Retriever—Example 1						
Score (3.5)	0.000	0.000	0.000	0.149	0.035	0.000
Score (4o)	0.333	0.670	0.000	0.187	0.051	0.000
Question	前処置で排便が必要な検査はありますか。					
	[Are there any tests that require bowel evacuation as a preparation?]					
Reference	ガリウム腫瘍/炎症シンチグラフィです。					
	[This is a gallium scintigraphy for tumors and inflammation.]					
Answer (3.5)	文中には排便が必要な検査についての記載はありません。したがって、前処で排便が必要な検査は特に言及されていません。					
	[There is no mention in the text of any studies that require bowel evacuation. Therefore, no such studies are specifically mentioned as part of the preparation.]					
Answer (4o)	文脈内には、前処置として排便が必要であると明記された検査は記載されていません。					
	[No study in the context is explicitly described as requiring bowel evacuation as a part of the preparation.]					
Vector Retriever—Example 2						
Score (3.5)	0.000	0.000	0.000	0.383	0.082	0.000
Score (4o)	0.000	0.570	0.570	0.229	0.068	0.000
Question	心筋血流シンチグラフィの前処置について説明してください。					
	[Please explain the pretreatment procedures for myocardial perfusion scintigraphy.]					
Reference	Tcの前処置は必要ないですが、Tlでは3時間の絶食が必要です。					
	[No pretreatment is necessary for Tc, whereas a three-hour fast is required for Tl.]					
Answer (3.5)	心筋血流シンチグラフィの前処置には、以下の項目が含まれます。—イソジンによる甲状腺ブロックが望ましい。 (10滴/日を2日前から)—検査前は閉眼安静を15分以上 これらの前処置が行われることで、心筋血流シンチグラフィの正確な結果を得るための準備が整えられます。					
	[Preparatory procedures for myocardial perfusion scintigraphy include the following:—Thyroid blocking with Isodine is recommended (10 drops/day, starting two days before the examination)—Rest with eyes closed for at least 15 min prior to the examination. These preparatory steps help ensure accurate results in myocardial perfusion scintigraphy.]					
Answer (4o)	心筋血流シンチグラフィ (201TlCl) の前処置について、文脈に基づいて説明します。 文脈には具体的な前処置に関する記載がありませんでした。そのため、心筋血流シンチグラフィの前処置についての詳細は、文脈外の情報を参照する必要があります。					
	[The pretreatment for myocardial perfusion scintigraphy (201TlCl) is explained based on the given context. However, no specific details regarding pretreatment were found in the context. Therefore, to obtain detailed information about the pretreatment procedures, it is necessary to refer to sources outside the provided context.]					
Vector Retriever—Example 3						
Score (3.5)	0.000	0.000	0.000	0.603	0.227	0.000
Score (4o)	0.333	0.570	0.000	0.604	0.154	0.000
Question	牛乳やチョコレー トなどの脂肪食を摂取すると画像が改善する場合がある検査はなんですか。またそれはなぜですか。					
	[Which imaging study may yield improved results after the ingestion of a fatty meal, such as milk or chocolate, and why?]					
Reference	心筋血流シンチグラフィです。薬剤の投与後に脂肪食を摂取することで、薬剤の肝胆道系からの排泄を促進させる効果があるためです。					
	[Myocardial perfusion scintigraphy. Consuming a fatty meal after the radiopharmaceutical is administered can promote its excretion through the hepatobiliary system.]					
Answer (3.5)	回答: 心筋脂肪酸代謝シンチ(123I-BMIPP)検査です。この検査では、心筋のエネルギー代謝を画像化することで心筋の状態を評価します。脂肪食を摂取することで、心筋の代謝が改善され、画像の品質が向上する場合があります。					
	[A: Myocardial fatty acid metabolism scintigraphy (123I-BMIPP). This test evaluates myocardial function by imaging its energy metabolism. Ingesting a fatty meal may enhance myocardial metabolism and improve image quality.]					
Answer (4o)	文脈に基づくと、牛乳やチョコレートなどの脂肪食を摂取すると画像が改善する場合がある検査は[胆道シンチ(99mTc-PMT)]です。 理由としては、脂肪食を摂取することで胆嚢収縮が促進され、胆汁の流れが良くなるため、胆道の通過や機能をより明確に評価できる場合があるからです。このような脂肪食の摂取は、胆道シンチにおいて胆汁の流れや胆道の異常を確認する際に役立つことがあります。					
	[In context, the test in which the ingestion of a fatty meal such as milk or chocolate may improve imaging results is hepatobiliary scintigraphy (99mTc-PMT). This is because fatty meals promote gallbladder contraction and enhance bile flow, potentially allowing for clearer assessment of biliary transit and function. Such fatty meal ingestion can be helpful in visualizing bile flow and detecting biliary abnormalities during the scan.]					
Vector Retriever—Example 4						
Score (3.5)	0.000	0.000	0.000	0.582	0.394	0.500
Score (4o)	0.333	0.000	0.500	0.342	0.030	0.000
Question	腹臥位と仰臥位で半量ずつ薬剤の投与が必要な検査は何ですか。					
	[Which study requires the radiopharmaceutical to be administered in two equal portions: one in the prone position and one in the supine positions?]					
Reference	肺血流/肺換気 シンチグラフィ99mTc-MAA/81mKrです。					
	[Pulmonary perfusion and ventilation scintigraphy using 99mTc-MAA and81mKr.]					
Answer (3.5)	腎静態シンチグラフィ(99mTc-DMSA)					
	[Static renal scintigraphy (99mTc-DMSA)]					
Answer (4o)	文脈内には、腹臥位と仰臥位で半量ずつ薬剤を投与する検査についての記載はありません。そのため、該当する検査は文脈からは特定できません。					
	[Since the context does not mention any study in which the radiopharmaceutical is administered in equal portions in the prone and supine positions, the specific study cannot be identified.]					
Vector Retriever—Example 5						
Score (3.5)	0.444	0.000	0.000	0.595	0.424	0.667
Score (4o)	0.889	0.890	0.570	0.568	0.088	0.400
Question	血糖値のmg/dLからmmol/Lへの換算係数を教えてください。					
	[What is the conversion factor for blood glucose levels from mg/dL to mmol/L?]					
Reference	18 mg/dL = 1 mmol/Lです。					
	[18 mg/dL = 1 mmol/L.]					
Answer (3.5)	血糖値のmg/dLからmmol/Lへの換算係数は、18.02です。					
	[The conversion factor for blood glucose from mg/dL to mmol/L is 18.02.]					
Answer (4o)	文脈には血糖値の換算係数に関する情報が含まれていません。そのため、一般的な知識としてお答えします。 血糖値をmg/dLからmmol/Lに換算するには、値を**18で割ります**。 例えば、100 mg/dLの血糖値は、100 ÷ 18 = 5.56 mmol/Lとなります。 逆に、mmol/Lからmg/dLに換算する場合は、値に**18を掛けます**。					
	[The context does not include information about the conversion factor for blood glucose levels, so I will provide a general explanation: To convert blood glucose levels from mg/dL to mmol/L, divide the value by 18. For example, a blood glucose level of 100 mg/dL is 100 ÷ 18 = 5.56 mmol/L. Conversely, to convert from mmol/L to mg/dL, multiply the value by 18.]					

This table highlights examples in which the retrieved content was judged to have low relevance to the question, regardless of the retrieval method used (vector-based or hybrid). Each row presents each evaluation metric, a user question, the corresponding gold-standard reference answer, and outputs generated by GPT-3.5 and GPT-4o. All questions, reference answers, and generated outputs were originally written in Japanese. The English translations shown in brackets [] are provided solely to aid understanding

These examples illustrate how insufficient retrieval accuracy—even when using strong language models—can result in hallucinated or contextually inappropriate answers

These behavioral differences between the models are consistent with previous findings, suggesting that a more capable foundation model tends to respond more cautiously when insufficient contextual information is available [21].

## Discussion

In this study, we developed an RAG system using Japanese institutional manuals for nuclear medicine imaging and evaluated its performance through manual assessment by radiological technologists and automated scoring using LLMs via RAGAS. Consequently, the configuration that combined GPT-4o with hybrid retrieval achieved the highest overall scores in both the human and automated evaluations, confirming its effectiveness as an optimal RAG system configuration.

This finding highlights the significant impact of the combination of the retriever and generator models on the output quality. In particular, when general-purpose vector models are used for domain-specific content, such as in Japanese medical manuals, hybrid retrieval methods that incorporate BM25 may perform more effectively [22].

Relatively large chunk sizes were used to ensure that the description of each application remained within a single chunk. As a result, the system was able to generate appropriate answers to technical questions related to image processing, such as “Explain the procedure for DaTQUANT analysis,” “Describe the process for generating QGS and QPS images in myocardial stress testing,” and “Explain the FDG image processing method for myocardial studies.” However, the use of large chunk sizes may have contributed to a reduction in retrieval precision [23, 24]. In the future, it will be necessary to explore optimization of chunk size to further improve retrieval accuracy, as well as to consider approaches such as retrieving smaller chunks and then including adjacent chunks in the prompt.

As shown in Tables 8 and 9, analysis of failure cases in both answer generation and retrieval revealed several limitations, including insufficient information in the manuals, incomplete retrieval coverage, and the difficulty of handling unstructured data such as figures and tables. Although foundation models with strong general language generation capabilities tend to be more robust to noise and include only the necessary information in their responses, it remains challenging to consistently generate appropriate answers to all questions. In particular, when retrieval was inadequate, hallucinations were frequently observed in responses generated by GPT-3.5. This poses a potential safety risk in the medical domain, highlighting the need for verification mechanisms and reliability indicators in future applications.

When evaluating the individual questions, the correlation between human ratings and RAGAS scores was not necessarily high. In particular, the correlation for the GPT-4o-mini was relatively low. An analysis of questions wherein GPT-3.5 and GPT-4o-mini showed divergent evaluations revealed that these were often questions requiring short answers, such as naming specific items or determining whether something was necessary. In such cases, the output from the GPTs tended to include additional contextual references to the source documents or question-wording, and GPT-4o-mini tended to penalize these additions more harshly than the other models or human evaluators. This likely contributes to the lower correlation. However, for questions requiring longer and more descriptive answers, both humans and the GPTs provided consistently high ratings of semantically accurate outputs. These observations suggest that the correlation coefficients vary depending on the question type.

Nonetheless, the overall ranking of the RAG system configurations was consistent between the human evaluations and the LLM-based automated evaluation metrics provided by the RAGAS. Thus, although the RAGAS has limitations in scoring individual answers, it can serve as a useful indicator for comparing system-level performance. Accordingly, the RAGAS may help reduce the burden of human evaluation, especially in large-scale system comparisons or during early-stage development. However, in this study we adopted only RAGAS as a representative “LLM-as-a-judge” approach and did not assess the systems with custom prompts or compare RAGAS with other automated evaluation frameworks; these comparative validations remain tasks for future work.

Additionally, traditional string-based metrics, such as ROUGE and Levenshtein distance, tend to undervalue semantically correct answers when alternative vocabulary or syntactic structures are used. In some cases, these metrics showed opposite trends to those of the human evaluators. These results support the importance of adopting LLM-based evaluation approaches that account for semantic understanding, particularly in specialized fields, such as medicine.

As shown in Table 7, the Friedman test revealed significant differences in average scores among the three human evaluators. In particular, the evaluator who was involved in constructing the question dataset showed a different scoring trend compared to the others, suggesting that this involvement may have influenced their evaluation. The remaining two evaluators had expertise in nuclear medicine and medical physics, respectively, and differences in professional backgrounds may have also contributed to score variability. However, as Fig. 2 also illustrates, this trend does not indicate a consistently harsher or more lenient scoring bias in any single direction. As shown in Table 10, the Fleiss’ kappa coefficient was approximately 0.4, indicating moderate agreement. While the evaluations for individual questions were generally consistent, some variability in interpretation was observed at the overall level. This may reflect structural factors such as the diversity of medical terminology, differences in interpretation of evaluation criteria, and variation in domain expertise [25]. However, because only three evaluators participated in this study, the data are insufficient to fully examine how their differing backgrounds may have influenced the scores, even though some variability in judgment is inevitable. Accordingly, in evaluations of natural language generation within the medical domain, it is not only essential to clarify evaluation standards and ensure alignment of understanding among evaluators, but also to secure a diverse and sufficiently large evaluator pool.

**Table 10:** Fleiss’ kappa scores for inter-rater agreement among the three human evaluators

	Total score answer (Hybrid/4o)	Total score answer (Hybrid/3.5)	Total score answer (Vector /4o)	Total score answer (Vector /3.5)	Total score retriever (Hybrid)	Total score retriever (Vector)
Fleiss’ Kappa	0.364	0.388	0.412	0.588	0.397	0.436

As noted earlier, even when RAG is employed, a non-negligible number of incorrect answers persist, meaning that current systems cannot yet be regarded as fully trustworthy; a similar pattern has been reported in RAG studies targeting other medical domains [26]. For real-world deployment, therefore, multi-layer safety architectures are required in addition to accuracy improvements achieved through model or architecture selection. Examples include interface designs that explicitly display the source passages retrieved—such as Google’s NotebookLM (https://notebooklm.google/)—and mechanisms for continuously collecting and analyzing low-confidence queries. Moreover, for examination items with poor retrieval performance, the source manuals themselves may need revision because human interpretation could otherwise become difficult. Because the present study is based on a single evaluation involving 100 cases, continuous performance monitoring and rigorous assessment of information-source quality—along with ongoing exploration of advanced model architectures—will be indispensable before routine clinical use.

The results of this study suggest that the RAG-based system may be a useful tool for supporting clinical decision-making in specialized domains. However, further validation is needed to assess the generalizability of these findings, including the use of models from other vendors. External validation was not conducted in this study, as both the corpus used as the knowledge source for retrieval and the evaluation dataset were designed based on the specific context of our institution, including internal documents and specialized terminology, making replication at other institutions currently difficult. Nevertheless, we have provided detailed descriptions of the system architecture, prompt design, and evaluation methods, with the aim of facilitating future validation at external sites.

## Conclusions

In this study, we developed a RAG system using Japanese institutional manuals for nuclear medicine imaging and evaluated its performance using expert human ratings and automated scoring with LLMs.

The combination of GPT-4o and hybrid retrieval achieved the highest evaluation scores. Although the agreement between human ratings and automated scoring was limited, the results suggest that the RAGAS may serve as a useful metric for the relative comparisons of RAG configurations.

Future studies should focus on improving the response accuracy and reliability of automated evaluation by refining the chunk design and optimizing prompt strategies.

## Supplementary Information

Below is the link to the electronic supplementary material.

Supplementary file1 (DOCX 46 KB)

Supplementary file2 (PDF 940 KB)

## Data Availability

The institutional manuals used in this study are internal documents of Shiga University of Medical Science Hospital. However, they are available from the corresponding author upon reasonable request.

## Code availability

The study did not involve new model development. Code for ensuring reproducibility can be provided upon reasonable request.
