# Supplementary material for: Evaluation of a retrieval-augmented generation system using a Japanese Institutional Nuclear Medicine Manual and large language model-automated scoring
Source: Radiol Phys Technol. 2025 Jul 19;18(3):861–76. doi: 10.1007/s12194-025-00941-y (PMC12339626; doi:10.1007/s12194-025-00941-y)
Supplement: Supplementary file 1 — Supplementary file1 (DOCX 46 KB) [file 12194_2025_941_MOESM1_ESM.docx]

**Supplementary Appendix**

Article title: Evaluation of a Retrieval-Augmented Generation System Using a Japanese Institutional Nuclear Medicine Manual and Large Language Model-Automated Scoring

Journal: Radiological Physics and Technology

Authors: Yusuke Fukui*, Yuhei Kawata, Kazumasa Kobashi, Yukihiro Nagatani, Harumi Iguchi

Corresponding author: fyusuke@shiga-med.ac.jp

**Table of contents:**

Section S1: Supplementary document

Section S2: Supplementary tables

**Section S1: Supplementary document**

Among the RI examination manuals used in this study, the manual for cerebral blood flow scintigraphy is provided as an example in a separate file titled *Supplementary Document.pdf*.

**Section S2: Supplementary tables**

Table S1. Questions and correct answers

Table S2. List of Python libraries used

Table S3. List of models used

Table S4. Results of Wilcoxon signed-rank tests (Hybrid/3.5)

Table S5. Results of Wilcoxon signed-rank tests (Hybrid/4o)

**Table S1. Questions and correct answers**

|  | Question | Reference answer | Category |
| --- | --- | --- | --- |
| 1 | 腎動態シンチグラフィの撮像時間を教えて下さい。 | 利尿なしは30分。利尿ありは60分です。 | K |
| 2 | 腎動態シンチグラフィの撮像範囲を教えて下さい。 | Dynamic撮像は腎臓上縁から膀胱下端までが入るように剣状突起から恥骨くらいにします。 | K |
| 3 | 腎静態シンチグラフィの摂取率の画像作成はどのように行いますか。 | DMSA・FULL・EMPTYを同時に選択し、[DMSA]アプリケーション起動します。年齢・身長・体重を入力し[OK]し、左腎をROIで囲み[Proceed]、右腎をROIで囲み[Proceed]し[Uptakein%Dose]が算出されていることを確認します。最後にCaptureColorにて保存します。 | S |
| 4 | 腎静態シンチグラフィは1と２のどちらの検出器を使用しますか。 | 検出器2を使用します。 | K |
| 5 | MAG3のレノグラムの画像のLayoutを教えてください。 | 0～2分はDisplayframetimeを[14seconds]、表示を[8×1]に設定します。2分以降はDisplayframetimeを[2min]、表示を[6×2]に設定します。利尿レノ場合は2分以降のDisplayframetimeを[4min]、表示を[7×2]に設定します。 | S |
| 6 | 腎動態シンチグラフィで患者が尿意をあればどうしますか。 | Scanを止めずに患者さんだけ寝台から降りてもらい、排尿後は再度寝台にのぼり、排尿後イメージを撮像します。 | A |
| 7 | 利尿剤の投与タイミングはどのように決定されますか。 | 泌尿器科医師が決定します。 | A |
| 8 | リンパ管シンチグラフィで準備する薬剤の放射能はいくつが目安ですか。 | 50～100Mbqが目安です。 | K |
| 9 | リンパ管シンチグラフィの検査時最大で何分後の撮影がありますか。 | 90分後です。 | K |
| 10 | リンパ管シンチグラフィの投与薬剤はどのように準備したら良いですか。 | 1mlのシリンジを用意し、シリンジに[プール]のシールを貼ります。キュリメーターにてバイアルの放射能(MBq)を測定し放射能(MBq)からシリンジに50～100MBqになるくらい抜き取ります。針を注射時に使用するもの(26Gくらい)に変えシリンジシールドを装着します。これは左右で２本用意します。 | S |
| 11 | リンパ管シンチグラフィで足を揉まないのは何科ですか。 | 形成外科です。 | A |
| 12 | 婦人科のセンチネルリンパ節シンチグラフィの薬剤は何を準備しますか。 | テクネフチン酸キットとメジテック(メジフィジックス)またはウルトラテクネ(富士フィルムRIファーマ)をしようします。カテラン針を装着した4本のシリンジを準備します。投与量は各約25-30Mbqで合計１００MBqです。 | K |
| 13 | 脊髄腔シンチグラフィの必要物品を教えてください。 | 薬剤、スパイラル針、圧測定菅、キシロカイン、スピッツ(髄液を採取する場合)、濾紙、穴あき滅菌シーツやドレープ、滅菌ガーゼ、創傷用粘着ドレッシング、消毒液(ポピドンヨードなど)、ハイポ清拭タオル、鉗子、滅菌手袋、モニター、ビニール袋、平オムツ、マジックペンです。 | S |
| 14 | 副腎髄質シンチグラフィの使用薬剤は何ですか。 | ミオMIBG123I注射液(111)（富士RIファーマ）です。 | K |
| 15 | 副腎髄質シンチの撮像したSPECT画像の画像作成処理について説明して下さい。 | 骨シンチのSPECT画像作成と同様です。SPECT-CTの場合は、Fusion(Ax・Sag・Cor)をVolumeで作成します。 | S |
| 16 | 131I-Adosterolの検査は何が目的ですか。 | 副腎皮質の検査で、クッシング症候群の病変部の診断や原発性アルドステロン症の鑑別が目的です。 | K |
| 17 | 副腎皮質シンチの投与薬剤と投与量を教えて下さい。 | 使用薬剤は131I-Adosterol、投与量は37MBqです。 | K |
| 18 | 副腎皮質シンチは薬剤投与後何分後に撮像しますか。 | ４日と7日後に撮像します。 | K |
| 19 | 肺血流シンチグラフィの斜位の角度はいくつですか。 | RAO、LAOともに45°です。 | K |
| 20 | 肺血流シンチグラフィで肺にROIを囲い解析する際は,何を選択して行いますか。 | 「QuantPerfusionAnalysis」を選択します。 | S |
| 21 | 肺血流シンチグラフィでシャント率を確認する場合はどのように撮影すれば良いですか。 | 投薬後速やかに撮像をします。コリメータはLEHRで[RLShunt]のプロトコールを使用します。 | S |
| 22 | 腹臥位と仰臥位で半量ずつ薬剤の投与が必要な検査は何ですか。 | 肺血流/肺換気シンチグラフィ99mTc-MAA/81mKrです。 | S |
| 23 | 肺血流のMAAの薬剤投与は当院ではどのように行っていますか。 | 重力効果軽減のために腹臥位と仰臥位で半量ずつ投与します。 | K |
| 24 | 肺血流のMAAで検査に必要な投与量はどの程度ですか。 | 185MBqです。 | S |
| 25 | MAAでシャント率測定を測定する際はどのようにしたら良いですか。 | コリメータはLEHRを使用し、薬剤投薬後速やかに[RLShunt]のプロトコールにて撮像を行います。 | K |
| 26 | 肺血流と肺換気シンチのLungAnalysisAladdinでの処理について説明してください。 | ANT/POST・RAO/LPO・RLAT/LLAT・RPO/LAOの4つを同時に選択し、[LungAnalysisAladdin]アプリケーションを起動し[QuantPerfusionAnalysis]を選択します。その後Post画像の肺野のROIを囲い、同様にAnt画像の肺野のROIも囲います。左右差を表示した画像が作成されるのでCaptureColorにて保存します。次に[Vent/Perf]アイコンをクリックし[POST/RPO/RLAT/RAOandRef]を選択し濃度・サイズを調整しCaptureColorにて保存します。最後に[LPO/LLAT/LAO/ANTandRef]を選択しCaptureColorにて保存します。 | S |
| 27 | MAAでのシャント率はどの程度が正常ですか。 | シャント率15%未満が正常となります。 | S |
| 28 | 肺血流/肺換気シンチグラフィでシャント率測定が必要な場合になぜ2部屋利用する必要があるのですか。 | シャント率測定のコリメータはLEHR、Kr/MAAの撮像はELEGPを利用し、検査途中でコリメータの交換作業を行わず効率よく検査を進めるためです。 | K |
| 29 | IMPによる脳血流の定性検査の送信画像はなんですか。 | 3Plane、Ax、Sag、Cor、iSSPDecreseGLB、iSSPTOMODecreseGLB(Ax)、iSSPTOMODecreseGLB(Sag,Cor)Z-GraphDecreaseGLB(脳内と精神科は+2）です。 | S |
| 30 | 脳血流シンチグラフィの統計解析処理に用いるFALCOMは何色のアプリケーションですか。 | 定性検査は赤色です。 | K |
| 31 | DaTScanの処理に関して、Bolt法でSBRを算出する画像は減弱補正が必要ですか。 | 減弱補正が必要です。 | S |
| 32 | 脳血流シンチグラフィのSPECT撮影時の検出器の距離を教えてください。 | 当院では回転半径14cm以下を推奨しています。 | K |
| 33 | GraphPlot法のDynamic撮像の患者位置は何を目安にしますか。 | PAと脳底を目安に撮像を行います。 | A |
| 34 | 脳血流シンチグラフィの123I-IMP定性検査の画像作成においてMultiFrameGridはどうように設定しますか。 | Axは5×4,Sag5×4、Corは5×5程度になります。 | S |
| 35 | GraphPlot法にて行う場合ルートはどちらでもよいですか。 | ボーラス性を担保するため必ず右腕からです。 | A |
| 36 | 脳血流の定量検査の際に行う動脈採血は何分後ですか。 | 静注10分後です。 | K |
| 37 | DaTQUANT解析の方法ついて説明してください。 | DaTQUANT解析①はNormalDBを選択し[Uptake]に画面右下のチェックを入れる。[ShowROI]にチェックを入れ、FrameGridを調整し少し拡大しRAINBOW表示にします。最後にCaptureColorにて保存します。集積がない患者は濃度の調整をします。DaTQUANT解析②は[Studyinfo]の中のGE-IRAC(吸収補正有のNormalDataBase)を選択し、[ShowROI]のチェックを外しScrolタブにて範囲を調整し、少し拡大してから画面右下の画像にROIを付けます。[StudyInfo]をOKしRAINBOW表示でCaptureColorにて保存します。 | S |
| 38 | BoltSBR解析では吸収補正なしのデータを利用しますか。 | 吸収補正ありのデータを利用します。 | S |
| 39 | CalibSBR解析では吸収補正なしのデータを利用しますか。 | はい。 | S |
| 40 | 一日で行う脳血流の負荷定量検査で、負荷剤はいつ投与しますか。 | 撮影開始後２０分です。 | K |
| 41 | 脳神経受容体シンチグラフィの撮像タイミングはいつですか。 | 20分と3時間後です。 | K |
| 42 | 脳血流検査では何MBqの薬剤を使用しますか。 | ECDでは400MBqまたは600MBq、IMPによる定性検査では167MBq、GraphPlot法は167MBq、ARG法は111MBq、DTARG法では222MBqです。 | K |
| 43 | 脳血流の定性検査は薬剤投与後何分に撮像を開始しますか。 | 薬剤投与後15分後です。 | K |
| 44 | 当院で使用する脳血流シンチグラフィの負荷薬剤はなんですか。 | アセタゾラミド（ダイアモックス）です。 | K |
| 45 | 脳血流定量検査のSeeMap解析の処理方法を教えて下さい。 | NonScaleを選択し、[SeeMap]を起動する。次に1stStudyに前回(Rest)のデータ、2stStudyに今回(Stress)のBinalyDataを選択し、Next→Excute→Saveで保存する。 | S |
| 46 | 唾液腺シンチグラフィの負荷のタイミングは何分ですか。 | 15分後にレモン負荷を行います。 | K |
| 47 | アルコールが含まれた薬剤を使用する検査はなんですか。 | 副腎皮質シンチグラフィとドパミントランスポーターシンチグラフィです。 | K |
| 48 | 前日に面線源を作成する必要がある検査は何ですか。 | リンパ管シンチグラフィです。 | K |
| 49 | 薬剤のpre撮像が必要な検査は何ですか。 | 甲状腺（Tc摂取率）、腎動態シンチグラフィ、腎静態シンチグラフィです。 | K |
| 50 | 当院でInを使用する検査は何がありますか。 | ソマトスタチン受容体シンチグラフィと骨髄シンチグラフィ、脊髄腔シンチグラフィがあります。 | K |
| 51 | 心血流シンチグラフィでSPECT撮影の際の、検出器の距離を教えてください。 | 当院では回転半径24cm以下を推奨しています。 | K |
| 52 | BMIPPを用いた心筋脂肪酸代謝シンチでは、心電図同期は必要ですか。 | 心電図同期は不要です。 | S |
| 53 | Heart Risk View解析においてStressのデータの名称は何に変更しますか。 | 「1st」です。 | K |
| 54 | 心筋血流シンチグラフィの前処置について説明してください。 | Tcは前処置の必要はないですが、Tlでは３時間の絶食です。 | S |
| 55 | 牛乳やチョコレートなどの脂肪食を摂取すると画像が改善する場合がある検査はなんですか。またそれはなぜですか。 | 心筋血流シンチグラフィです。薬剤の投与後に脂肪食を摂取することで、薬剤の肝胆道系からの排泄を促進させる効果があるためです。 | A |
| 56 | 心筋血流シンチグラフィ負荷検査の安静時はいつ薬剤を投与しますか。 | 負荷時投与から3時間後に薬剤投与します。 | K |
| 57 | 心筋の負荷検査のQGSとQPS画像の作成手順を説明してください。 | FBPのCARDIACSPECTフォルダを選択し、[QGS/QPS]アプリケーション起動します。QGS解析(Stress)は[QuantitativeGatedSPECT]にチェックが入っていることを確認し、画像上のプルダウンからSTRESS_FBP(Gated)になっていることまた、ROIが心筋に適切に重なっていることを確認します。その後CaptureColornonheaderで保存します。QGS解析(Rest)は[QuantitativeGatedSPECT]にチェックが入っていることを確認し、画像上のプルダウンからRest_FBP(Gated)になっていることまた、ROIが心筋に適切に重なっていることを確認します。その後CaptureColornonheaderで保存します。QPS解析は[QuantitativePerfusionSPECT]を選択しCaptureColornonheaderで保存します。Cine(Stress)画像作成[QuantitativeGatedSPECT]を選択し画面上の[View]をクリック、画面上のプルダウンがSTRESS_FBPになっていることを確認しDynamicCaptureで保存します。 | S |
| 58 | ピロリン酸シンチグラフィの対象疾患はなんですか。 | 心アミロイドーシスの診断が目的で、AL型心アミロイドーシスとATTR型心アミロイドーシスの鑑別が行われます。 | K |
| 59 | ピロリン酸シンチグラフィで投与3時間後の撮像が必要なのはどんなときですか。 | 投与後1時間後の画像でH/Clratioが1.3から1.4程度のように心筋の集積が曖昧な場合です。 | A |
| 60 | ブルズアイ画像の作成には何のアプリケーションを使用しますか。 | 「GEWomap」です。 | K |
| 61 | 99mTc-TFを用いた心筋血流の負荷検査で送信する画像をおしえてください。 | static、OSEMColor、FBPColor、FBPBW、QGSstress、QGSrest、QPS、Cine(Stress)、Womap、HeartRiskViewです。 | K |
| 62 | 心筋交感神経シンチで心筋に取り込みが無い場合の画像処理はどうしたら良いですか。 | [Myovayion]での3断面やブルズアイ画像は作成せずに[VolumeMI]にて心臓が存在すると思われる部分のAxとCorを作成します。 | A |
| 63 | ブルズアイ画像の作成時に「Rest counts Hide washout map?」とメッセージは出ますか。またそのときはどうすれば良いですか。 | 「Rest counts Hide washout map?」とメッセージがでたらOKを押して下さい。 | S |
| 64 | 心筋交感神経シンチグラフィの解析時にROIをどこに置きますか。 | 心臓と上縦隔、肺野です。 | S |
| 65 | ピロリン酸シンチグラフィの解析時にROIをどこに置きますか。 | 心筋部とその対側です。 | S |
| 66 | 67Gaの腫瘍シンチの撮像は投与から何日後ですか。 | 投与から2日後です。 | K |
| 67 | ガリウムに使用されるコリメータは何を使用しますか。 | コリメータはMEGPです。 | K |
| 68 | 前処置で排便が必要な検査はありますか。 | ガリウム腫瘍/炎症シンチグラフィです。 | K |
| 69 | ソマトスタチン受容体シンチグラフィの前処置は何ですか。 | オクトレオチド酢酸塩等のソマトスタチンアナログによる治療が行われている場合は、検査前に休薬することが望ましいです。 | S |
| 70 | ソマトスタチン受容体シンチグラフィの検査目的はなんですか。 | ソマトスタチン受容体の発現の画像化、ならびに神経内分泌腫瘍(NET)の局在及び転移診断を行うためです。 | K |
| 71 | ソマトスタチンは標識後何時間以内の投与が必要ですか。 | 標識後6時間以内です。 | K |
| 72 | ソマトスタチンのSPECT-CTの撮像範囲はどこですか。 | 範囲の記載はなく、検査ごとに画像を見て確認します。 | K |
| 73 | ソマトスタチンシンチのコリメータは何ですか。 | MEGPです。 | K |
| 74 | ガリウムシンチは前処置が必要ですか。 | 検査前日に下剤と排便が必要です。 | K |
| 75 | 骨シンチグラフィの使用薬剤を依頼科ごとに教えて下さい。 | 呼吸器内科と泌尿器科、乳腺一般外科は99mTc-MDP555MBq(フジRIファーマ)です。その他は99mTc-HMDP(クリアボーン)555MBq(日本メジフィジックス)です。 | A |
| 76 | 消化管出血の使用薬剤はなんですか。 | プールシンチ注（日本メジフィジックス）です。 | K |
| 77 | 消化管出血シンチグラフィでは最大何時間後の撮像を行いますか。 | 24時間後です。 | K |
| 78 | 蛋白濾出シンチグラフィでは最大何回撮像しますか。 | 最大で5分後,15分後,30分後,45分後,60分後,3時間後,6時間後,24時間後に撮影するため8回です。 | K |
| 79 | 蛋白濾出シンチグラフィでCTは使用しますか。 | 出血・濾出が確認できたタイミングでSPECT-CTを撮像する場合があります。 | K |
| 80 | HSADではどの程度の出血から診断できますか。 | 微量の出血(0.01～0.1ml/min)でも検出可能です。 | K |
| 81 | GI-BONEを使用する際の発注するべきRI製剤はHMDPかMDPのどちらですか。 | HMDPです。 | K |
| 82 | GI-BONEの解析時の吸収補正は何ですか。 | Gaussianです。 | S |
| 83 | 甲状腺ブロックはどの程度しますか。 | 2日前から10滴/日で行います。 | K |
| 84 | 甲状腺シンチで、亜急性甲状腺炎の臨床像はどのようになりますか。 | 亜急性甲状腺炎では集積が見られないことがほとんどです。 | A |
| 85 | ヨード甲状腺シンチグラフィの検査前に摂取してはいけないものはなんですか。 | ヨードを含む海藻類や昆布加工品です。 | K |
| 86 | 甲状腺の解析時ROIの形はどれですか。 | ROITypeはPOLYGONを選択します。 | S |
| 87 | 甲状腺の摂取率の解析処理の説明をお願いします。 | Xeleris上で、Full、Empty、Staticの3データを選択し、[GEThyroidA100]アプリケーションを起動します。次にTc-99mThyroidUptakeボタンを押し、ROITypeはPOLYGONを選択します。各場所にROIをとり保存します。 | S |
| 88 | 甲状腺腫瘍シンチは投与後何分後に撮像しますか。 | 投与後５分後と３時間後です。 | K |
| 89 | 甲状腺腫瘍シンチの患者のポジショニングを教えて下さい。 | 体位はHead First Supineとし、首の下にクッションを入れ下顎挙上し頭部を固定します。 | S |
| 90 | 副甲状腺シンチグラフィの送信画像の一覧をお願いします。 | Static、FusedAx、FusedCor、FusedSag、NMAx)、NMCor、NMSag、NMMIP、CT、DoseReportです。 | K |
| 91 | 肝受容体シンチグラフィの定量値であるHH15・LHL15の正常値を教えてください。 | HH15は0.50から0.57、LHLはから0.96です。 | K |
| 92 | GSAの撮像タイミングはいつですか。 | 薬剤投与と同時にDynamic撮像を行います。 | K |
| 93 | 肝受容体シンチグラフィの患者体位は何ですか。 | Foot First Supineです。 | S |
| 94 | GSAの撮像範囲を教えて下さい。 | 心臓～肝臓が入るように(鎖骨～腸骨稜くらい)で設定します。 | K |
| 95 | 肝GSAの検査のTrans axialデータはなぜ必要なのですか。 | SPECT画像とfusionすることで診断を容易にします。また当院では消化器医師がVINCENTを用いて術前の解析を行うため必要となります。 | A |
| 96 | 胆道シンチではDynamic以外に何を撮像しますか。 | Dynamic以外にStatic、SPECTを撮像します。 | K |
| 97 | 血糖値のmg/dLからmmol/Lへの換算係数を教えてください。 | 18mg/dL＝1mmol/Lです。 | K |
| 98 | アミロイドPET検査の投与後の待機時間と撮像時間はどの程度ですか。 | 待機時間は90分、撮像時間は１時間です。 | K |
| 99 | 心筋を対象としたFDGの画像処理について説明してください。 | AW上で、FDGCardiacのデータを選択しCardIQFusionPETを選ぶ。FusionQCを選択し、軸設定をSA/VLA/HLA方向とする。自動で軸が設定されない場合は手動で軸設定を行う。上部の任意軸設定ボタンクリックからSA,HLA,VLAに角度の調整を行う。Colorは画面上部の▼をクリックし、カラーパレットボタンよりRainbow表示にする。Batchから各FOVは25で保存する。断面はそれぞれObliqueなら(例Axi→Oblique)にしないと保存出来ない。画像左上の文字上で右クリックし、フュージョンなし画像を選択し同様に保存する。次にCTCardiac Static Cardiacのデータを選択し、Xelerisに転送する。Xelerisにて、Myovationアプリケーションを起動し心臓3断面処理時と同様の処理を行う。 | S |
| 100 | FDG検査の前処置について教えてください。 | 5時間の絶食と運動制限、検査直前の排尿です。心臓の検査においては12時間絶食です。 | S |

**Table S2. List of Python libraries used**

| Library Name | Version | Purpose |
| --- | --- | --- |
| ^langchain-core^ | ^0.30^ | Core components for building language model applications |
| ^langchain_openai^ | ^0.2.0^ | OpenAI integration for LangChain |
| ^langchain-community^ | ^0.3.0^ | Community-contributed tools and integrations for LangChain |
| ^langchain-text-splitters^ | ^0.3.0^ | Utilities for splitting text into manageable chunks for processing |
| ^langchain-chroma^ | ^0.1.4^ | Integration with Chroma vector store for retrieval-based applications |
| ^ragas^ | ^0.2.8^ | Evaluation framework for Retrieval-Augmented Generation (RAG) systems |
| ^rank-bm25^ | ^0.2.2^ | Implementation of the BM25 ranking algorithm for information retrieval |
| ^SudachiDict-full^ | ^20250129^ | Full Japanese dictionary used by Sudachi for morphological analysis |
| ^SudachiPy^ | ^0.6.10^ | Japanese tokenizer and morphological analyzer |

This table provides a list of Python libraries employed in the development of the system, including their version numbers and brief descriptions of their purposes. These libraries are primarily used for text processing, retrieval-augmented generation (RAG), and Japanese language analysis.

**Table S3. List of models used**

| Model name in the text | Version | Purpose |
| --- | --- | --- |
| ^gpt-3.5^ | ^gpt-3.5-turbo-0125^ | Used to generate and evaluate answers |
| ^gpt-4o^ | ^gpt-4o-2024-11-20^ | Used to generate answers |
| ^gpt-4o-mini^ | ^gpt-4o-mini-2024-07-18^ | Used to evaluate answers |
|  |  |  |

This table summarizes the large language models (LLMs) employed in the study, specifying the model names, versions, and their respective purposes (e.g., answer generation, evaluation).

**Table S4. Results of Wilcoxon signed-rank tests (Hybrid/3.5)**

| Comparison | Wilcoxon Statistic | Adjusted p-value (Holm) | Effect size \|r\| |
| --- | --- | --- | --- |
| eval1 vs eval2 | 91.5 | p<0.01 | 0.340 |
| eval1 vs eval3 | 171.0 | 0.460 | 0.074 |
| eval2 vs eval3 | 175.0 | p<0.001 | 0.363 |

This table presents the results of Wilcoxon signed-rank tests conducted to compare the evaluation scores among three evaluators (eval1, eval2, and eval3) under the Hybrid/3.5 condition. The post-hoc tests were performed because the Friedman test indicated significant differences. Multiple comparisons were adjusted using the Holm method. The table reports the adjusted p-values and effect sizes (|r|).

The mean evaluation scores indicated that eval2 tended to assign lower scores (mean: 0.617) compared to eval1 (0.730) and eval3 (0.763).

Evaluator eval1 is a certified nuclear medicine technologist with 25 years of experience in nuclear medicine, eval2 is a radiological technologist with 5 years of experience in nuclear medicine, and eval3 is a medical physicist with 15 years of experience in nuclear medicine.

**Table S5. Results of Wilcoxon signed-rank tests (Hybrid/4o)**

| Comparison | Wilcoxon Statistic | Adjusted p-value (Holm) | Effect size \|r\| |
| --- | --- | --- | --- |
| eval1 vs eval2 | 41.0 | p<0.01 | 0.282 |
| eval1 vs eval3 | 311.5 | 0.382 | 0.087 |
| eval2 vs eval3 | 25.0 | p<0.001 | 0.407 |

This table presents the results of Wilcoxon signed-rank tests conducted under the Hybrid/4o condition. The post-hoc tests were performed because the Friedman test indicated significant differences. Multiple comparisons were adjusted using the Holm method. The table reports the adjusted p-values and effect sizes (|r|). The mean evaluation scores were 0.827 for eval1, 0.907 for eval2, and 0.817 for eval3.
