# Supplementary material for: Evaluation of a retrieval-augmented generation system using a Japanese Institutional Nuclear Medicine Manual and large language model-automated scoring
Source: Radiol Phys Technol. 2025 Jul 19;18(3):861–76. doi: 10.1007/s12194-025-00941-y (PMC12339626; doi:10.1007/s12194-025-00941-y)

脳血流シンチグラフィ
(123I-IMP)負荷定量：ARG 法）

虚血性脳血管障害、アルツハイマー病、痴呆、もやもや病、一過性脳虚血発作などの診断。虚血脳血流量の測定。

薬剤情報・前処置・撮像条件等

1. 使用薬剤

- ➢ 123I-IMP 111MBq ：商品名：パーヒューザミン注(111)（日本メジフィジックス）
- ➢ アセタゾラミド（ダイアモックス）：2バイアル

2. 投与量：111MBq

3. 前処置

- ➢ イソジンによる甲状腺ブロックが望ましい。（10滴/日を2日前から）
- ➢ 検査前は閉眼安静を15分以上

コリメーター：ELEGP

検査方法：SPECT

撮像開始時間：静注10分前に薬剤負荷。静注10分後動脈採血。静注25分後撮像開始。

撮像

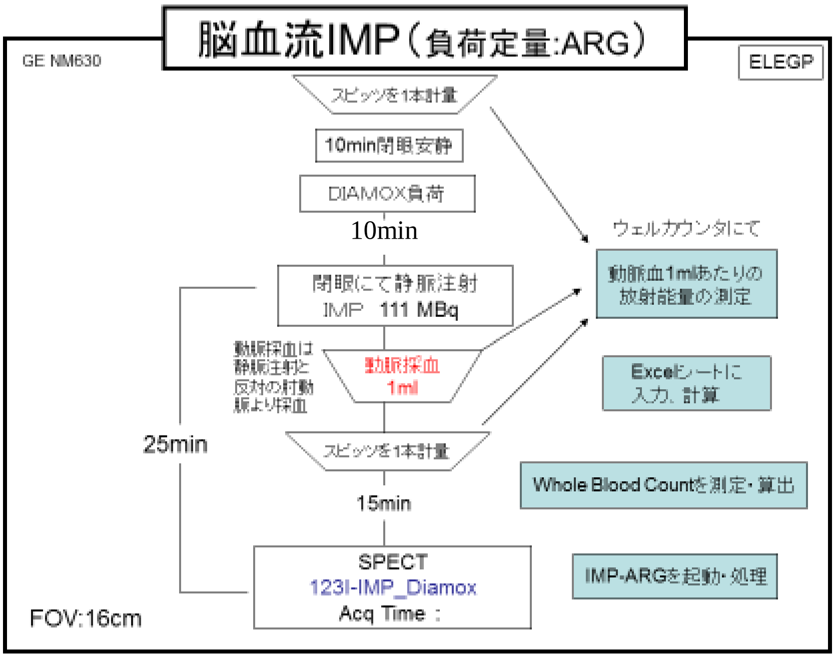

撮像

1. 検査前準備
   - ➢ ヘッドレストを装着
   - ➢ 処置室にて空スピッツの重量を測定

1) 蓋をせずに計測
2) 測定した重量はメモ用紙に記入

2. 必要物品の調達

1) 薬剤：IMPパーヒューザミン注（111）
2) 20mlシリンジ×2：生理食塩水を引く用とダイアモックス用
3) 注射針：20mlシリンジのキャップ替わり。余っている針でよい
4) 生理食塩水20ml×2：フラッシュ用とダイアモックス溶解用
5) アル綿×2：消毒用。静脈注射時と動脈採血時に使用
6) クロルヘキシジングルコン酸塩含浸綿×2：消毒用(アルコールかぶれがある人)
7) 翼状針21G or 23G：静脈ルート確保用
8) サーフロー22G or 24G：静脈ルート確保用
9) 三活栓（ニプロ針付き活栓）：確保した静脈ルートと生食入りシリンジ・薬剤をつなぐ
10) 延長チューブ500mm：サーフローと三活栓をつなぐ
11) プラネクタ：病棟患者など、点滴ですでにルートが確保されている人用。ルートと三活栓のコネクタの役割
12) 静脈止血布（ステプティ青）：静脈止血用。インジェクションパッド（ピンクの袋）を好む先生も
13) 動脈採血キット：動脈採血時に使用
14) 動脈血止血布（ステプティ赤）：動脈止血用
15) 駆血帯：静脈確保用。
16) テープ：静脈ルート確保時の針固定用
17) ストップウォッチ
18) キムタオル：手台汚れ防止用
19) ビニール袋：ゴミ捨て用
20) 手袋：感染予防
21) ダイアモックス　2バイアル
22) サチュレーションモニター
23) テルモ生食500ml
24) ポンプ用輸液セット
25) IV3000

3. 注射

1) 患者にベッドに寝てもらい、ベッドを注射がしやすい高さに上げる

2) 注射する側の上肢を手台にのせる

3) ダイアモックスの作成 (脳血管外科医師が施行)

① 生食20mlでダイアモックスを溶解し、シリンジに引く

4) 静脈ルート確保＆薬剤注入(脳血管外科医師が施行)

① 延長チューブ、三活栓、ポンプ用輸液セット、テルモ生食をつなぎ、生食で満たしておく

② サーフローでルート確保

③ サーフローと延長チューブをつなぐ

④ ダイアッモクス注入

⑤ 10分待機

⑥ RI薬剤注入

□□ストップウォッチスタート

□□15秒かけて注入

4. 動脈採血**(**薬剤投与**10**分後**)**

1) 静脈注射した腕とは反対側の腕で準備

2) 3分くらい前でDrにアナウンス

3) 時間になったら、動脈採血キッドにて、動脈採血

4) 動脈用止血布にて止血

5) 採血した動脈採血キッドに蓋をして、預かる

5. 動脈血測定

1) 採血したらできるだけ早く測定へ

2) 採血した注射から、空スピッツに動脈血を移す(大体1ml)

3) 動脈血入りスピッツの重量を測定

4) メモ用紙に記入

5) スピッツに蓋をする

6) ウェルカウンタにスピッツをセット

7) [Start]ボタンを押す

8) 測定が終了すれば、測定結果用紙を取り出す

6. 撮像**(**薬剤投与**25**分後**)**

➢ 通常IMPと同じ

処理

1. Whole Body Countの算出

1) ウェルカウンタにて測定した用紙を処置室から持ってくる

2) ARG用のExcelファイルを開く

➢ 初回の患者は新しいファイルを作成

➢ ２回目以降の患者はすでにファイルがあるので、新しいタブを作成して使用

3) 必要な値を入力し、Whole Body Countを算出

4) 依頼表・メモ用紙・測定結果用紙はホッチキスでまとめて保存

2. 加算データの作成

5) TOMO_rot1～TOMO_rot4を全て選択し、[DS sum MEW X3 251]アプリケーションを起動

6) Sum Planar → Select Start(1を選びOK) → Select End(10を選びOK) → OK → Save Data(名前はPlanar_TOMOのままでいい) → アプリを閉じる(×ボタン)

7) DS_SumPlanar360×2ファイルが作成される

3. 画像再構成

1) DS_SumPlanar360×2ファイルを選択し、[I123]を起動

2) 撮影範囲が欠けていないことを確認しProceed

3) Thickを2に設定(Sag、Axi、Corすべて)

4) 3断面の軸を合わせる(SagはAC-PCライン)

5) 範囲を欠けない程度に絞り(赤線)、中心軸も合わせる

6) SPECT OPTIONにて再構成条件を調整

7) File → Save＆Exit ➔ BRAIN SPECT、SNAP SHOTSファイルが作成される

4. ３**Plane**画像作成

1) BRAIN SPECTファイルを選択し、[GE BrainREV X3 130]アプリケーションを起動

2) 濃度を調整

3) Scrollタブにて範囲を調整

4) アノテーションを挿入(123I-IMP ARG Rest)

5) Pan and Zoomタブにて、サイズ・位置を調整

6) Capture Colorで保存

5. Ax・Sag・Cor画像作成

1) BRAIN SPECTファイルを選択し、[GE ARG x3 180]アプリケーションを起動

2) Creat CBF → パラメーター入力 ➔ OK

3) Ax画像作成

① Transversalを選択

② Window Levelを調整(30～50くらい) ➔ Proceed

- これ以降濃度をいじることはない

③ Number of Rowを4、Number of Colを5に設定 → exit

④ Scrollタブを開き範囲を調整

⑤ アノテーションを挿入(123I-IMP ARG rest)

⑥ Pan and Zoomタブを開き、サイズ・位置（前後上下）を調整

⑦ 頭頂部からの出力になっていることを確認

⑧ Capture Colorにて保存

⑨ Exit

4) Sag画像作成

- Ax画像と同様

5) Cor画像作成

- Ax画像と同様
- Multi Frame Gridを5×5程度に

6) File ➔ Save＆exit ➔ Yes

- GE_ARG_Results_Non Scaleが生成される

6. 統計解析処理**(3D-SSP＆NEVRO FLEXER)**

1) XelerisにてBRAIN SPECT - Transversal Obl、を選択し、GE_ARG_Results_Non Scale – Transversal CBFを選択 ➔ Export

2) 解析ソフト端末(Xeleris1横パソコン)にてExportフォルダを開く

3) 解析ソフト端末にてCドライブ内のARG_DATAフォルダを開く

4) 初回の患者：該当するアルファベットフォルダに患者ファイルを作成(名前_ID)

- ２回目以降の患者：該当するアルファベットフォルダに存在する患者ファイルを選択
- 患者ファイル内にExportフォルダ内にある患者データをコピー

5) コピーした患者データのファイル名を今日の日付に変更（2016.12.22）

6) [FALCON]を起動

7) 患者DICOMデータをドラック＆ドロップ

8) 3D-SSP解析

- 通常IMP定量と同じ

9) NEVRO FLEXER解析　＊安静定量の解析参照

① Non Scale を選択し、[NEVRO FLEXER]を起動(2 回目以降の患者は前回と今回のデータを選択)

② 1st Study に前回(Rest)のデータ・2st Study に今回(Stress)のデータを選択(初回の場合は1st Study に今回のデータを選択) → NEXT →はい

③ 3 断面を選択(何でもOK)(前回ある時は1 断面) → Execute(画面右下のSag 画像にて向きが正しいか確認してから) → OK → レポートが作成される

10)See Map解析

- 安静定量を過去に行っている場合は See Map解析も行う

① Non Scale を選択し、[See Map]を起動

② 1st Study に前回(Rest)のデータ・2st Study に今回(Stress)のBinaly Dataを選択

③ Next→Excute→Save

7. シリーズ番号を変更

- Falcon もしくはXeleris どちらかで

送信画像

- **3Plane**
- **Ax**

- **Sag**
- **Cor**
- **iSSP Decrease GLB**
- **iSSP TOMO Decrease GLB STDAx)**

画像一覧

Diamox（3Plane、Ax、Cor、Sag）

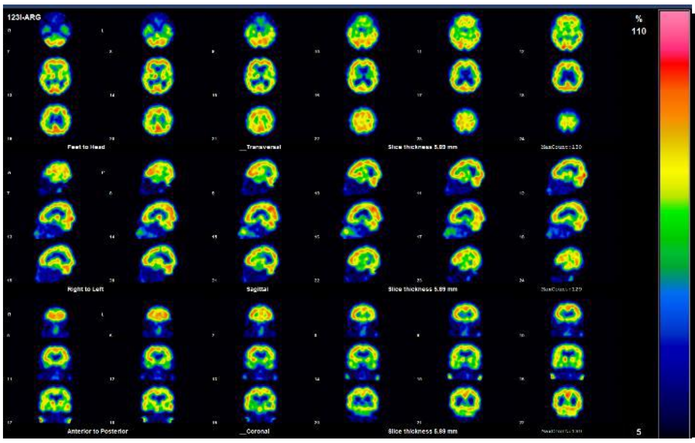

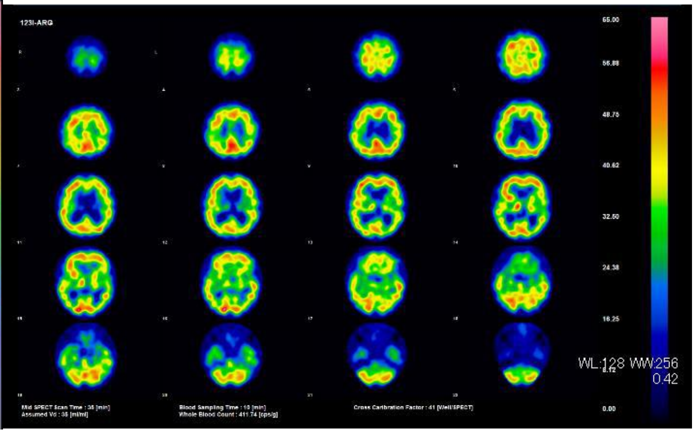

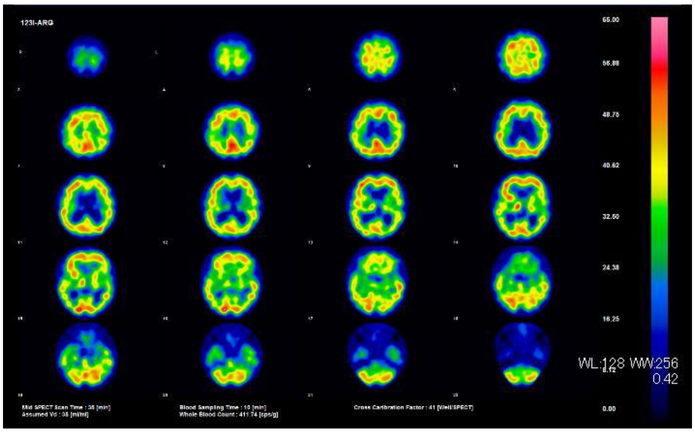

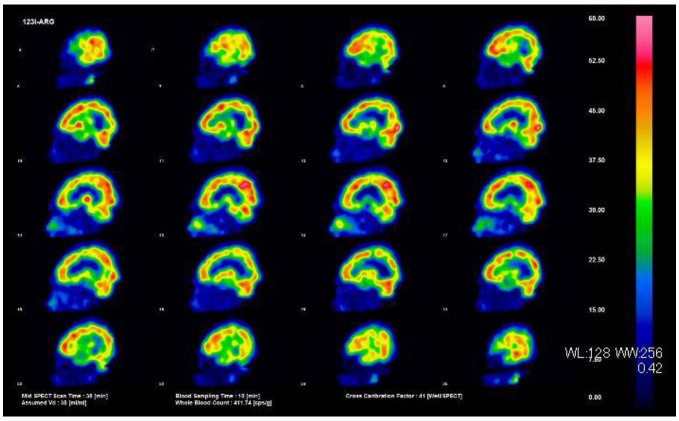

Diamox（iSSP、Tomo Ax Sag Cor）

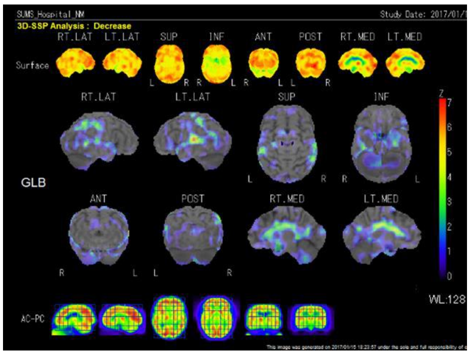

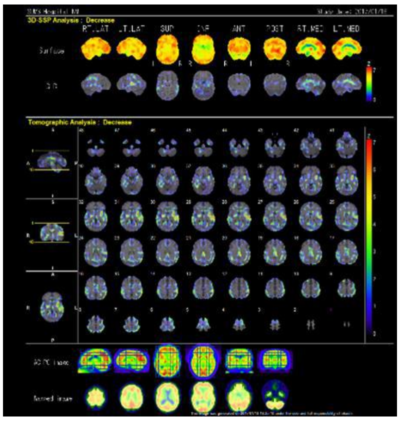

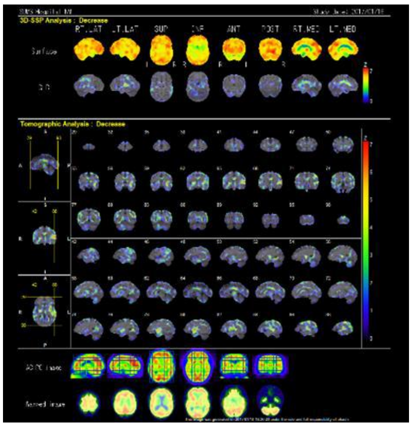

NEURO FLEXER

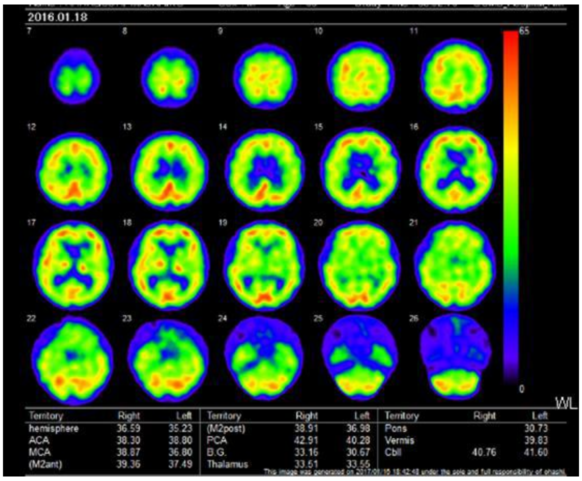

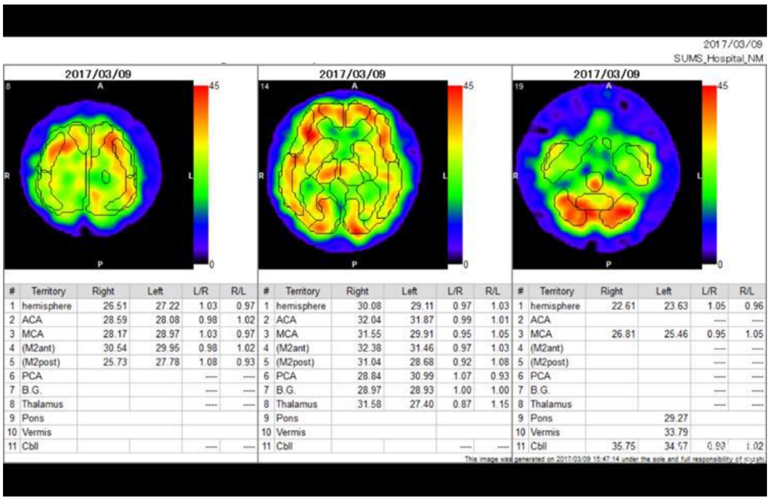

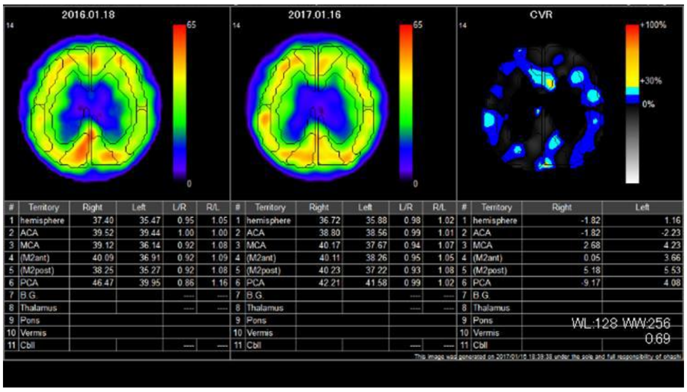

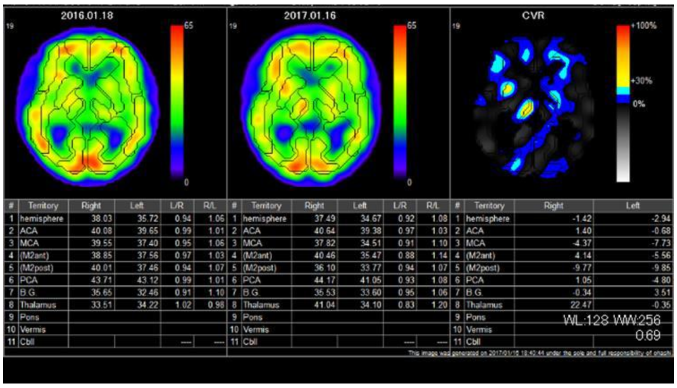

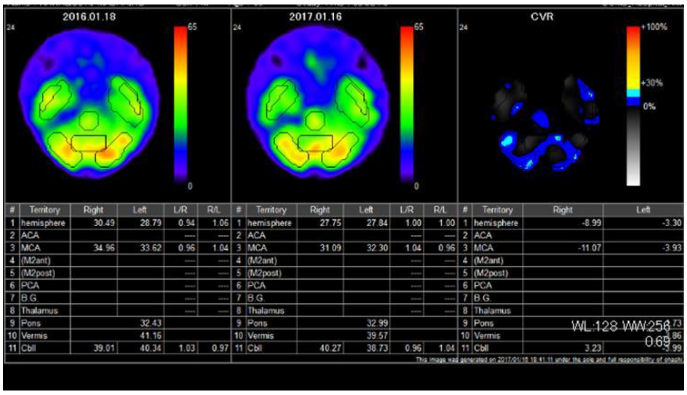

Supplement: Supplementary file 2 — Supplementary file2 (PDF 940 KB) [file 12194_2025_941_MOESM2_ESM.pdf]
